# Ischemia–Reperfusion Injury in Porcine Aortic Valvular Endothelial and Interstitial Cells

**DOI:** 10.3390/jcdd10100436

**Published:** 2023-10-19

**Authors:** Jennie H. Kwon, Miriam Atteya, Alekhya Mitta, Andrew D. Vogel, Russell A. Norris, Taufiek Konrad Rajab

**Affiliations:** 1 Department of Surgery, Medical University of South Carolina, Charleston, SC 29425, USA; kwonhye@musc.edu (J.H.K.); atteya@musc.edu (M.A.); 2 Department of Medicine and Cell Biology, Medical University of South Carolina, Charleston, SC 29425, USA; norrisra@musc.edu; 3 School of Medicine, University of South Carolina, Columbia, SC 29208, USA; alekhya.mitta@uscmed.sc.edu; 4 Division of Research, Alabama College of Osteopathic Medicine, Dothan, AL 36303, USA; vogela@acom.edu; 5 Division of Pediatric Cardiovascular Surgery, Arkansas Children’s Hospital, Little Rock, AR 72202, USA

**Keywords:** pediatric cardiac surgery, partial heart transplantation, congenital cardiac surgery, valve dysfunction, porcine, ischemia reperfusion injury, valvular endothelial cells, valvular interstitial cells

## Abstract

Ischemia–reperfusion injury (IRI) in the myocardium has been thoroughly researched, especially in acute coronary syndrome and heart transplantation. However, our understanding of IRI implications on cardiac valves is still developing. This knowledge gap becomes even more pronounced given the advent of partial heart transplantation, a procedure designed to implant isolated human heart valves in young patients. This study aims to investigate the effects of IRI on aortic valvular endothelial cells (VECs), valvular interstitial cells (VICs), and whole leaflet cultures (no separation of VECs and VICs). We employed two conditions: hypoxic cold storage reperfusion (HCSR) and normothermia (NT). Key markers, secreted protein acidic and cysteine rich (SPARC) (osteonectin), and inducible nitric oxide synthase (iNOS2) were evaluated. In the isolated cells under HCSR, VICs manifested a significant 15-fold elevation in SPARC expression compared to NT (*p* = 0.0016). Conversely, whole leaflet cultures exhibited a 1-fold increment in SPARC expression in NT over HCSR (*p* = 0.0011). iNOS2 expression in VECs presented a marginal rise in HCSR, whereas, in whole leaflet settings, there was a 1-fold ascent in NT compared to HCSR (*p* = 0.0003). Minor escalations in the adhesion molecules intercellular adhesion molecule (ICAM), vascular cell adhesion molecule (VCAM), E-selection, and P-selection were detected in HCSR for whole leaflet cultures, albeit without statistical significance. Additionally, under HCSR, VICs released a markedly higher quantity of IL-6 and IL-8, with respective *p*-values of 0.0033 and <0.0001. Interestingly, the IL-6 levels in VECs remained consistent across both HCSR and NT conditions. These insights lay the groundwork for understanding graft IRI following partial heart transplantation and hint at the interdependent dynamic of VECs and VICs in valvular tissue.

## 1. Introduction

Ischemia–reperfusion injury (IRI) remains highly relevant to orthotopic heart transplantation [1]. IRI can lead to early graft dysfunction, which primarily manifests as ventricular dysfunction, and prolonged ischemic times are associated with increased risk for early mortality among transplant recipients [1]. In the face of this association, IRI in cardiomyocytes is the subject of many studies. In contrast, the transplant biology of valvular tissue has only recently become of interest in the context of partial heart transplantation, a new transplant technique seeking to deliver growing heart valve implants [2,3,4,5,6,7,8]. Heart valves serve a hemodynamic function to ensure unidirectional blood flow, but they also maintain important biological functions such as growth and self-repair. These biological functions depend on cellular viability. Previously, we studied the cellular viability of rodent aortic valve grafts in cold preservation [9]. However, the contribution of subsequent reperfusion after storage in cold preservation remains unexplored. IRI may result in valve cell damage that affects the biological functions of heart valves. Understanding the characteristics of IRI in valves would therefore provide an initial context to explore strategies to mitigate the impact of IRI on the performance of partial heart allografts in vivo.

Our current study aims to characterize the pattern of IRI in porcine aortic valvular tissue.

## 2. Materials and Methods

### 2.1. Valve Dissection

**Figure 1:**
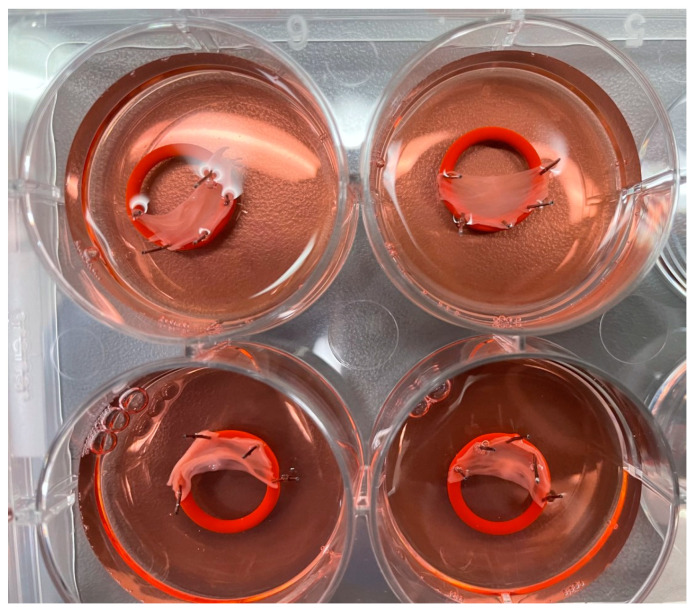
The photograph shows valve leaflets placed in a 6-well TC-treated culture plate.

Fresh porcine cardiac tissue was obtained from a slaughterhouse for cell culture experiments, and from the MUSC animal laboratory for tissue (whole leaflet) culture experiments to minimize warm ischemia time. All tissues were harvested immediately after animal death. Cardiotomy was performed, and individual valve leaflets of the aortic valves were isolated. Using three to four 0.1 mm dissection pins (Living Systems Instrumentation, Fairfax, VT, USA), the base of each leaflet was pinned to the edge of a 10.8 mm or 18.6 mm sterile silicone ring (Sigma, cat. No. Z50433 or Z504343, respectively, St. Louis, MO, USA) at a slight tension, leaving the free edge of the leaflet to face the center of the ring. Single leaflets were collected by cutting between the commissures of leaflets. For tissue culture, leaflets were specified as right coronary, left coronary, or non-coronary. Individual leaflets were placed separately in a 6-well TC-treated culture plate (Corning, cat. No. 3506, Corning, NY, USA). Each leaflet was covered with 10 mL of 4 °C Wisconsin Buffer (Global Transplant Solutions, Spartanburg, SC, USA) (Figure 1).

### 2.2. Valvular Endothelia Cell and Valvular Interstitial Cell Isolation

Culture media for valvular interstitial cells (VICs) were prepared in Dulbecco’s Modified Eagle Media (DMEM) and supplemented with 10% fetal bovine serum, 1% GlutaMax (Gibco, cat. no. 35050061, Waltham, MA, USA), 1% MEM non-essential amino acids (Gibco, cat. no. 11140050), 1% mL penicillin/streptomycin, and 0.4% Amphotericin B. Culture media for valvular endothelial cells (VECs) and for tissue culture were additionally supplemented with 2% heparin (1000 U/mL).

Valve leaflets were placed in a 35 mm dish and incubated at 37 degrees Celsius with 3 mL of cold Liberase solution for 5–10 min. After the incubation period, each part of the leaflet surface was gently swabbed, and the swab was immersed into a collagenase solution to dislodge the cells. The cell suspension/collagenase solution was transferred to a 15 mL conical tube, and 3 mL of VEC media was added to the tube. The solution was centrifuged at 1000 rpm for 5 min, washed, and then plated into T75 flasks for VICs and 35 mm culture dishes for VECs. All experiments on isolated VECs and VICs were performed at 90% confluence.

### 2.3. Hypoxia Induction

Cell and tissue cultures were exposed to 6 h of cold hypoxic storage in University of Wisconsin (UW) buffer. Media were aspirated from culture wells, which were then washed with cold 4 °C UW three times. Culture dishes were placed in an airtight chamber (STEMCELL Technologies, cat. no. 27310, Vancouver, BC, Canada). Hypoxic conditions were induced using displacement with nitrogen gas flush for a 90 s interval followed by 90 s of rest and another 120 s interval of nitrogen flush. Cultured cells in the hypoxia chamber were stored at 4 degrees Celsius for 6 h. This process was repeated for RC and LC leaflets in tissue culture. Leaflets labeled NC were control leaflets and were stored at 37 °C for 6 h.

### 2.4. Media Preparation for Reperfusion

After 6 h of culture in hypoxic conditions in University of Wisconsin buffer, the buffer in each well was harvested for analysis. An aliquot of the prepared media was warmed in a 37 degrees C water bath, and 10 mL was added to each well, replacing the buffer. Media were replaced every 2–3 days for cell culture and every 7–14 days for tissue cultures.

### 2.5. RNA Isolation and Molecular Quantification

#### 2.5.1. Cell Culture

To prepare RNA lysate, 2-Mercaptoethanol (BME) was added to a solution of RLT lysis buffer in a 1:100 dilution ratio and set aside. After the supernatant was collected from each well, about 500 μL of the RLT-BME was added. The floor of each well was scraped with a clean 200 μL pipette tip. RNA lysate was collected, and RNA was isolated using the RNeasy Kit. RNA content was quantified using RT-qPCT for the following genes: glyceraldehyde-3-phosphate dehydrogenase (GAPDH), platelet endothelial cell adhesion molecule 1 (PECAM1), smooth muscle actin, alpha 2 (ACTA2), hypoxia-inducible factor-1 (HIF1A), secreted protein acidic and cysteine rich (SPARC), and vascular endothelial growth factor (VEGF). Interleukin (IL)-6 and IL-8 levels were measured using an ELISA.

#### 2.5.2. Tissue Culture

Each leaflet was centrally placed in between two glass coverslips and flash frozen by firmly pressing a dry ice pellet on top of the coverslip for 5 s. The coverslips were then gently separated, and the leaflet was transferred onto a 60 mm plate. In total, 700 μL of a BME + RLT solution combined in a 1:100 dilution ratio was added to this dish. The leaflet was cut into smaller pieces using scissors, and the solution was prepared as VIC RNA lysate using a tissue homogenizer and transferred in 1 mL aliquots into cryovials. The two coverslips were placed into a 60 mm dish with the side pressing the leaflet facing upward. A total of 600 mL of RLT lysis buffer was added to the dish, and a bent 10 μL pipette tip was used to scrape the coverslips to prepare the VEC RNA lysate. The resulting solution was transferred in 1 mL aliquots into cryovials. RNA lysate was then isolated using the RNease Kit. RNA content was quantified using RT-qPCR for the following genes: SPARC, inducible nitric oxide synthase (iNOS2), P-selectin, E-selectin, intercellular adhesion molecule (ICAM), and vascular cell adhesion molecule (VCAM).

### 2.6. Immunofluorescence of Fixed Cells in Falcon Four-Chamber Slides

An aliquot of VIC and VEC cell culture in media was added to 4-chamber falcon slides. The cells were allowed to adhere. The cell medium was aspirated, and the slide chambers were washed well with 400 μL of Dulbecco’s phosphate-buffered saline (DPBS). The cells were fixed in 250 μL of 4% PFA for 15 min. After this time, PFA was removed by washing the cells three times with DPBS. The slides were stored at 4 °C in the last wash of DPBS. The next day, DPBS was aspirated, and the cell chambers were initially blocked with normal goat serum for 15 min. A specific primary antibody for each CD31, von Willebrand factor (vWF), and α-SMA was added to each chamber while leaving one chamber as the negative control. The chambers were incubated at 4 °C overnight. Primary antibodies were aspirated the next day, and secondary antibodies diluent were added. These were left to incubate for 1–2 h in the dark before they were aspirated and washed with DPBS three times. Slides were placed on a rocker in the last wash while still covered to protect from light. Finally, the last DPBS wash was aspirated, and the cell chamber was sealed with coverslips and allowed to finish curing at 4 °C overnight. Immunostaining was analyzed the next day.

### 2.7. Primer Design for Polymerase Chain Reaction (PCR) Assays

To begin designing primers, the NCBI “Nucleotide” search engine was utilized to identify the gene of interest, specifically within the Sus scrofa species. The search was narrowed down to results ending with “mRNA” and having an Accession number commencing with “NM_”. Subsequently, the Accession number was inputted into the NCBI Primer-designing tool. Primer parameters were adjusted to a maximum PCR product size of 200, with primer melting temperatures set to a minimum of 59 °C, optimum of 62 °C, and maximum of 65 °C. Selected Primer pairs were separated by at least one intron, with a minimum length range of 200. Primer pair specificity checking parameters were adjusted according to the appropriate taxid of the organism. Primer pairs were meticulously selected based on a set of criteria, including PCR product size (70–200 base pairs), primer length (18–22 nucleotides), and a GC% content (50–60%). Additionally, primers were chosen to bind near the 3′ end of the gene and were validated for low self- and 3′-complementarity to minimize primer–dimer formation. Primer specificity was further confirmed using Primer-BLAST, ensuring no off-target binding in the genome. In this study, specific primers were designed for VECs and VICs to check the expression of essential genes. For VECs, primers were selected for PECAM1, iNOS2, ICAM, VCAM, E-selectin, and P-selectin. For VICs, primers were designed targeting ACTA2, runt-related transcription factor 2 (RUNX2), SPARC, also known as osteonectin, and collagen type I alpha 1 chain (COL1A1). Additionally, primers for GAPDH, heat shock protein family B (Small) member 1 (HSPCB), and DNA topoisomerase II beta (TOP2B) were designed to serve as housekeeping genes, providing a stable reference for normalization and ensuring the reliability and accuracy of gene expression data in the subsequent PCR assays.

## 3. Results

**Figure 2:**
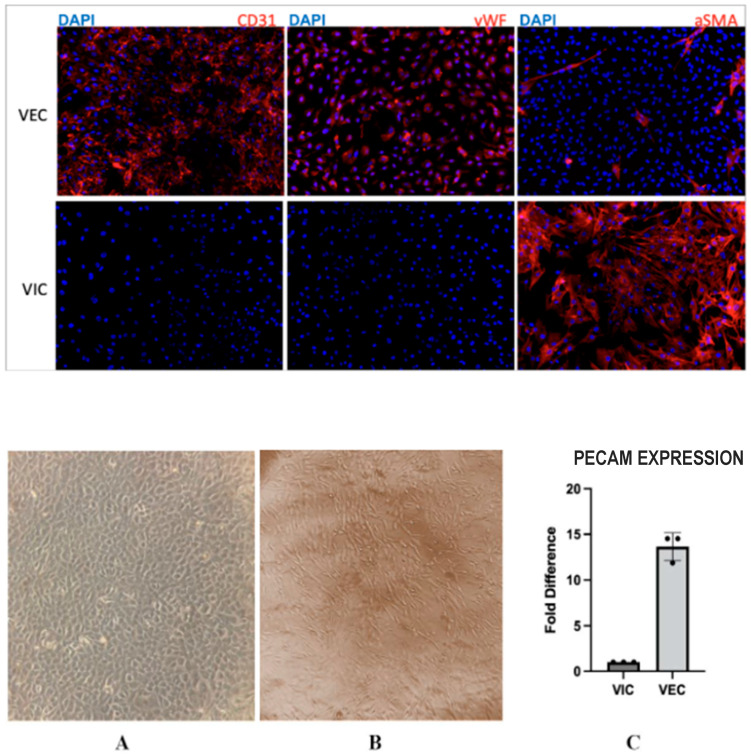
The **upper panels** show a comparison of protein expression, indicating the successful isolation of VECs and VICs via immunohistochemistry. CD31 and vWF are expressed in VECs, while absent in VICs. aSMA is expressed in VICs, while absent in VECs. The **lower panels** show morphology of VECs in culture compared to VICs. VECs conform to a cobblestone morphology in vitro (**A**), while VICs are more spindle-shaped (**B**). The bar graph (**C**) shows quantification of the gene encoding platelet and endothelial cell adhesion molecule (PECAM1) using RT PCR. There is an increase in PECAM1 expression in VECs compared to VICs. Black circles indicate individual data points.

**Figure 3:**
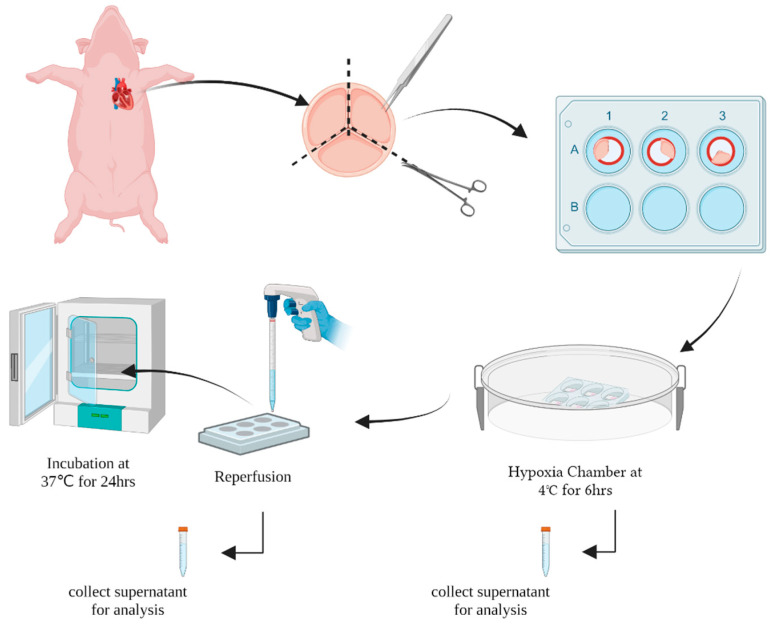
The schematic shows the experimental design for simulation of hypoxia-cold storage conditions. Image created by BioRender.com. Black dotted lines indicate cuts in the valve.

The successful separation of VECs and VICs was demonstrated via immunohistochemistry staining (Figure 2) and further confirmed using RTqPCR (Figure 3). Isolated VECs consistently showed increased expressions of PECAM1 and vWF, both of which uniquely reside on the endothelium, whereas VICs, although heterogenous in nature containing fibroblasts, myofibroblasts, and smooth muscle cells, consist mostly of fibroblasts, meaning that they have an increased expression of alpha smooth muscle actin (aSMA) [10]. VECs also showed themselves to be cobblestone shape at microscopy compared to the spindle shape appearance of VICs.

**Figure 4:**
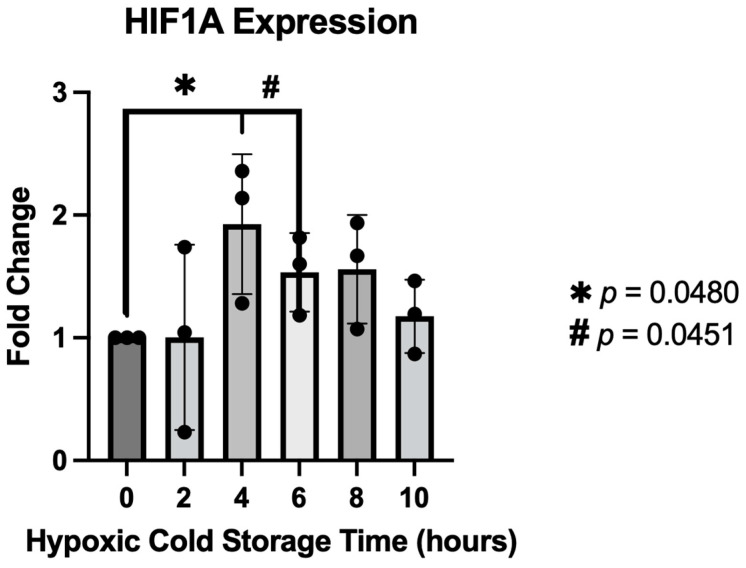
The bar graph shows hypoxia-inducible factor 1 expression at various hypoxia cold storage times. A significant increase in HIF1A expression is seen at 4 and 6 h of hypoxic conditions compared to control cultures not exposed to hypoxia. Black circles indicate individual data points.

The simulation of ischemic conditions was tested by comparing the expression of HIF1A at various stages of hypoxic cold storage. A significant increase in HIF1A expression was observed at four (*p* = 0.048) and six hours (*p* = 0.045) post-ischemic induction (Figure 4). These data correlate with former studies that determined the standard duration of cold storage allowed before transplantation.

**Figure 5:**
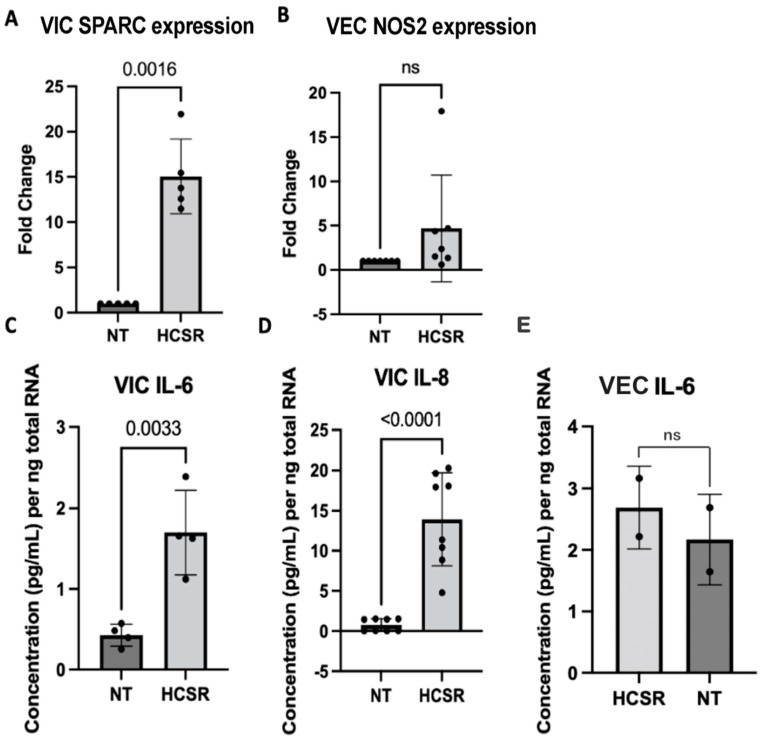
These graphs show findings in isolated cell culture. Graphs A and B show mRNA quantification by RT PCR. Graphs C and D indicate ELISA IL-6 and IL-8 cytokine expression in VICs, normalized to RNA content. Graph E indicates ELISA IL-6 cytokine expression normalized to RNA content. Under hypoxic cold storage conditions, VICs show an increased expression of osteonectin (SPARC), *p* = 0.0016 (**A**). No significant change was found in VECs for inducible nitric oxide synthase gene (NOS2) in VECs (**B**). VICs were found to induce the release of IL-6 and IL-8 under hypoxic cold storage conditions compared to control (**C**,**D**). VECs showed no significant (ns) change in IL6 release under these conditions (**E**). Black circles indicate individual data points.

**Figure 6:**
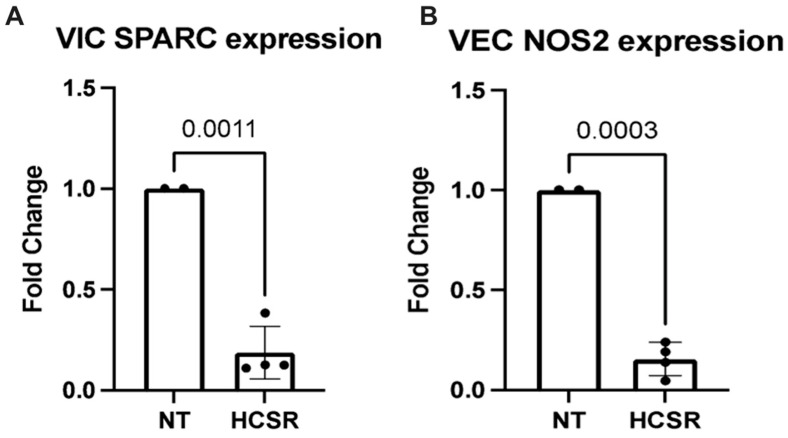
These graphs show findings in whole valvular leaflet cultures. Graphs A and B show mRNA quantification via PT PCR. Under hypoxic cold storage conditions, VICs show an increased expression of osteonectin (SPARC), *p* = 0.0011 (**A**). VECs were also found to show increased expression of inducible nitric oxide synthase gene (NOS2), *p* = 0.0003 (**B**). Black circles indicate individual data points.

Ischemia reperfusion injury was simulated in the isolated colonies of VECs and VICs (Figure 5), as well as whole valvular leaflets (Figure 6). Control samples were kept in a nutrient-rich medium under normothermic conditions 37C (NT). Experimental samples were initially preserved in University of Wisconsin buffer at cold storage conditions for 6 h, and then buffer was replaced with nutrient-rich media for 24 h to simulate hypoxia cold storage reperfusion (HCSR). RNA was extracted from each colony, and the presence of different protein expressions was analyzed. VICs consistently had a larger RNA yield when compared to VECs under HCSR. For further analysis, VECs RNA yield was corrected to reflect such change.

To investigate the effects of ischemic injury, the study identified osteonectin, also known as SPARC, as a measure of oxidative stress in VIC and iNOS2 in VECs. In the isolated cell culture, an increase in SPARC expression was observed in the HCSR culture in comparison to the NT culture by 15-fold (*p* = 0.0016). However, in the whole leaflet culture, an increase in SPARC expression was observed in the NT culture in comparison to the HCSR culture by 1-fold (*p* = 0.0011). In the isolated cell culture, iNOS2 expression was slightly increased in the HCSR culture in comparison to the NT culture, while the whole leaflet culture demonstrated an increase in the NT culture in comparison to the HCSR culture by 1-fold (*p* = 0.0003). The study also evaluated the expressions of ICAM, VCAM, E-selection, and P-selectin in the whole leaflet culture. The expression of ICAM, VCAM, E-selection, and P-selection was slightly increased in the HCSR culture in comparison to the NT culture for the whole leaflet cultures; however, these results were not statistically significant.

The release of inflammatory proteins was also measured in the supernatant. The concentration of Il-6 released by VICs was significantly increased under HCSR when compared to NT (*p* = 0.0033). A more significant pattern was seen in the expression of interleukin-8 (Il-8) (*p* < 0.0001). Contrary to expectations, the concentration of IL-6 in VEC supernatant was not significantly different between HCSR and NT conditions.

## 4. Discussion

Our study primarily aimed to delineate the IRI responses in VECs and VICs, examining them either as isolated entities or within the context of the entire leaflet during exposure to ischemic conditions. SPARC and iNOS2 are biomarkers typically expressed by cells under stress; elevated levels suggest tissue damage. Our findings highlight marked disparities in responses between isolated cells and whole leaflet cultures under HCSR. Specifically, isolated VICs under HCSR exhibited heightened SPARC expressions relative to those under normothermia. In contrast, SPARC levels in whole leaflet cultures were higher under normothermia than HCSR. A parallel trend was observed for iNOS2 in VECs, where a slight increase was observed in HCSR in isolated cell cultures, in contrast to only a one-fold increase in the NT culture compared to HCSR in the whole leaflet samples. These findings indicate that the pattern of ischemia–reperfusion injury displayed in isolated cell lines may be muted or even reversed in whole leaflet cultures subjected to the same environment, suggesting a dynamic interdependence between VECs and VICs. In cytokine analyses, VICs released significantly elevated amounts of IL-6 and IL-8 during HCSR, suggesting a more pronounced role for VICs in IRI. VECs displayed no notable variation in IL-6 levels under similar conditions. The observed differences might be influenced by the lower yield from VEC isolations compared to VICs, indicating the need for further investigations to solidify these conclusions.

Understanding ischemia–reperfusion injury in valvular tissue has become a topic of increased interest, especially in light of the current literature on partial heart transplant as a novel procedure for congenital valvular disease in the pediatric population [4,9,11]. The current treatment for valvular dysfunction involves valve replacement with a bioprosthetic, Ross pulmonary autotransplantation, or conventional orthotopic heart transplantation. A partial heart transplant would offer the option of transplanting only donor valves instead of a whole heart transplant. The procedure could decrease the duration of immunosuppression required, given that a mechanical counterpart could later replace the orthotopic donor valve in adulthood [4].

Therefore, understanding IRI in valvular tissues holds immense clinical relevance. Mitigating these injuries could drastically improve outcomes following procedures like partial heart transplants. Characterization of cellular injury is the first step toward understanding the underlying mechanisms, a process that paves the way for more targeted therapeutic strategies, ultimately enhancing post-operative recovery and the long-term success of these procedures.

Our findings particularly spotlight the VICs as central players in IRI within valvular tissues. The marked involvement of VICs is evident from their heightened SPARC expressions and their elevated release of cytokines such as IL-6 and IL-8 during HCSR, suggesting that these cells might serve as prime targets for interventions.

Contrary to expectations, there was a marked discrepancy in responses between isolated cells and whole leaflet cultures under HCSR. Specifically, isolated VICs under HCSR exhibited heightened SPARC expressions relative to those under normothermia, whereas the opposite was observed in whole leaflet cultures. Such findings were also observed, although to a lesser extent, in iNOS2 expression in VECs versus whole leaflets. These findings indicate that the pattern of ischemia–reperfusion injury displayed in isolated cell lines may be muted or even reversed in whole leaflet cultures subjected to the same environment, suggesting a dynamic interdependence between VECs and VICs.

The relationship between VECs and VICs has been explored in previous research, revealing notable interactions. A study by Hjortnares et al. suggests that VICs directly inhibit VEC osteogenic differentiation, as expressions of osteocalcin, osteopontin, and Runx2 were directly reduced when VECs were cultures in the presence of VICs as opposed to when they are absent when both cultures were exposed to factors that induced osteogenic differentiation through an endothelial-to-mesenchymal transformation (EndMT) process [12]. Comparably, under hypoxic conditions, a review by Katsi et al. suggests that the angiogenic factors secreted by VICs might act on endothelial cells to promote their proliferation and the formation of new vessels [13]. As in these instances, a similar interaction may be implicated in ischemia reperfusion injury that reduces expressions of SPARC and iNOS2 in the whole leaflet cultures observed in this study.

In this study, the observed that variations in cytokine expression between VICs and VECs might be influenced by the lower yield from VEC isolations compared to VICs. The separation of VECs from VICs was achieved using a cold press method, as previously described. Future research might consider exploring alternative isolation methods to enhance yield. Additionally, it is crucial to note a potential variable: the site-specific properties of VECs. A review by Dev and Lacerda highlighted that VECs in the aortic valve exhibit different responses to shear flow depending on whether they are located on the aortic or ventricular sides of the valve leaflets. Specifically, VECs on the aortic side express fewer genes associated with inhibiting calcification [14]. This study did not adjust for these site-specific differences, which might have resulted in a disproportionate isolation of cells from one site over the other.

Future studies could explore the exact molecular signaling events in VICs during IRI and any potential crosstalk with VECs. Given the intricate interdependence between VECs and VICs we have observed, understanding this interaction may reveal synergistic strategies to combat ischemic injuries more effectively. By prioritizing VICs in this research trajectory, we aim to make impactful strides in enhancing the durability and function of valvular tissues post-ischemia, significantly benefiting patient outcomes in the long run.

## 5. Conclusions

This study characterizes injury patterns in VECs and VICs subjected to IRI. Understanding the characteristics of IRI in VECs and VICs will provide the first step toward finding ways to mitigate the impact of IRI in partial heart transplant graft preservation. For example, IRI may be mitigated using monoclonal antibodies, pharmaceutical drugs, and metabolic aids. While our data suggest certain markers may be factors in IRI, further research must be conducted to modulate these markers. Importantly, outflow valve dysfunction rarely occurs after orthotopic heart transplants [15,16,17,18,19].

## Data Availability

Data are contained within the article.

## References

[1] Singh T.P., Cherikh W.S., Hsich E., Harhay M.O., Hayes D., Perch M., Potena L., Sadavarte A., Zuckermann A., Stehlik J. (2022). The International thoracic organ transplant registry of the international society for heart and lung transplantation: Twenty-fifth pediatric heart transplantation report-2022; focus on infant heart transplantation. J. Heart Lung Transplant. Off. Publ. Int. Soc. Heart Transplant..

[2] Rajab T.K. (2020). Evidence-based surgical hypothesis: Partial heart transplantation can deliver growing valve implants for congenital cardiac surgery. Surgery.

[3] Sherard C., Bisbee C., Konsek H., Kang L., Turek J.W., Rajab T.K. (2023). Partial Heart Transplantation in Adult Cardiac Surgery. Innovations.

[4] Sherard C., Atteya M., Vogel A.D., Bisbee C., Kang L., Turek J.W., Rajab T.K. (2022). Partial heart transplantation can ameliorate donor organ utilization. J. Card. Surg..

[5] Hill M.A., Kwon J.H., Gerry B., Kavarana M., Nadig S.N., Rajab T.K. (2021). A Simplified Model for Heterotopic Heart Valve Transplantation in Rodents. J. Vis. Exp. JoVE.

[6] Bishara K., Kwon J.H., Hill M.A., Helke K.L., Norris R.A., Whitworth K., Prather R.S., Rajab T.K. (2023). Characterization of Green Fluorescent Protein in Heart Valves of a Transgenic Swine Model for Partial Heart Transplant Research. J. Cardiovasc. Dev. Dis..

[7] Konsek H., Sherard C., Bisbee C., Kang L., Turek J.W., Rajab T.K. (2023). Growing Heart Valve Implants for Children. J. Cardiovasc. Dev. Dis..

[8] Skidmore S., Hill M.A., Bishara K., Konsek H., Kwon J.H., Brockbank K.G.M., Rajab T.K. (2023). Morbidity and Mortality of Heterotopic Partial Heart Transplantation in Rodent Models. J. Cardiovasc. Dev. Dis..

[9] Kwon J.H., Hill M.A., Gerry B., Morningstar J., Kavarana M.N., Nadig S.N., Rajab T.K. (2021). Cellular Viability of Partial Heart Transplant Grafts in Cold Storage. Front. Surg..

[10] Bertipaglia B., Ortolani F., Petrelli L., Gerosa G., Spina M., Pauletto P., Casarotto D., Marchini M., Sartore S. (2003). Cell characterization of porcine aortic valve and decellularized leaflets repopulated with aortic valve interstitial cells: The VESALIO Project (Vitalitate Exornatum Succedaneum Aorticum Labore Ingenioso Obtenibitur). Ann. Thorac. Surg..

[11] Rajab T.K., Ochoa B., Zilinskas K., Kwon J., Taylor C.L., Henderson H.T., Savage A.J., Kavarana M., Turek J.W., Costello J.M. (2023). Partial heart transplantation for pediatric heart valve dysfunction: A clinical trial protocol. PLoS ONE..

[12] Hjortnaes J., Shapero K., Goettsch C., Hutcheson J.D., Keegan J., Kluin J., Aikawa E. (2015). Valvular interstitial cells suppress calcification of valvular endothelial cells. Atherosclerosis.

[13] Katsi V., Magkas N., Antonopoulos A., Trantalis G., Toutouzas K., Tousoulis D. (2020). Aortic valve: Anatomy and structure and the role of vasculature in the degenerative process. Acta Cardiol..

[14] Deb N., Lacerda C.M.R. (2021). Valvular Endothelial Cell Response to the Mechanical Environment—A Review. Cell Biochem. Biophys.

[15] O’Brien M.F., Stafford E.G., Gardner M.A., Pohlner P.G., McGiffin D.C. (1987). A comparison of aortic valve replacement with viable cryopreserved and fresh allograft valves, with a note on chromosomal studies. J. Thorac. Cardiovasc. Surg..

[16] Valente M., Faggian G., Billingham M.E., Talenti E., Calabrese F., Casula R., Shumway N.E., Thiene G. (1995). The aortic valve after heart transplantation. Ann. Thorac. Surg..

[17] Mitchell R.N., Jonas R.A., Schoen F.J. (1998). Pathology of explanted cryopreserved allograft heart valves: Comparison with aortic valves from orthotopic heart transplants. J. Thorac. Cardiovasc. Surg..

[18] Hill M.A., Kwon J.H., Gerry B., Hardy W.A., Walkowiak O.A., Kavarana M.N., Nadig S.N., Rajab T.K. (2021). Immune Privilege of Heart Valves. Front. Immunol..

[19] McVadon D.H., Hardy W.A., Boucek K.A., Rivers W.D., Kwon J.H., Kavarana M.N., Costello J.M., Rajab T.K. (2022). Effect of cardiac graft rejection on semilunar valve function: Implications for heart valve transplantation. Cardiol. Young.

